# *N*,4-Dimethyl­benzamide

**DOI:** 10.1107/S1600536811003527

**Published:** 2011-02-05

**Authors:** Jia-Ying Xu, Wei-Hua Cheng

**Affiliations:** a College of Chemical and Biological Engineering, Yancheng Institute of Technology, Yinbing Road No. 9 Yancheng, Yancheng 224051, People’s Republic of China; b Department of Chemical Engineering, Yancheng College of Textile Technology, Yancheng 224051, People’s Republic of China

## Abstract

In the crystal of the title compound, C_9_H_11_NO, mol­ecules are connected *via* inter­molecular N—H⋯O hydrogen bonds, forming a one-dimensional network in the *b*-axis direction. The dihedral angle between the amide group and the benzyl ring is 13.8 (2)°.

## Related literature

For the synthetic procedure, see: Lee *et al.* (2009 ▶). For bond-length data, see: Allen *et al.* (1987 ▶). ?show [softreturn]>
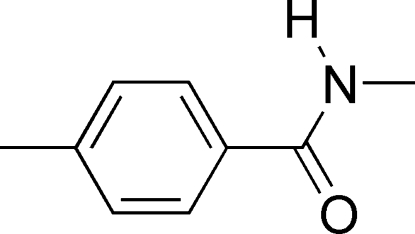


## Experimental

### 

#### Crystal data

C_9_H_11_NO
*M* *_r_* = 149.19
Monoclinic, 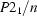

*a* = 6.7670 (14) Å
*b* = 9.946 (2) Å
*c* = 12.229 (2) Å
β = 92.63 (3)°
*V* = 822.2 (3) Å^3^
*Z* = 4
Mo *K*α radiation
μ = 0.08 mm^−1^
*T* = 293 K
0.30 × 0.20 × 0.10 mm

#### Data collection

Enraf–Nonius CAD-4 diffractometer
Absorption correction: ψ scan (North *et al.*, 1968 ▶) *T*_min_ = 0.977, *T*_max_ = 0.992
3362 measured reflections
1510 independent reflections
1062 reflections with *I* > 2σ(*I*)
*R*_int_ = 0.033
3 standard reflections every 200 reflections  intensity decay: 1%

#### Refinement

*R*[*F* ^2^ > 2σ(*F* ^2^)] = 0.050
*wR*(*F* ^2^) = 0.166
*S* = 1.01
1510 reflections
103 parameters
H-atom parameters constrained
Δρ_max_ = 0.20 e Å^−3^
Δρ_min_ = −0.15 e Å^−3^

### 

Data collection: *CAD-4 Software* (Enraf–Nonius, 1985 ▶); cell refinement: *CAD-4 Software*; data reduction: *XCAD4* (Harms & Wocadlo,1995 ▶); program(s) used to solve structure: *SHELXS97* (Sheldrick, 2008 ▶); program(s) used to refine structure: *SHELXL97* (Sheldrick, 2008 ▶); molecular graphics: *SHELXTL* (Sheldrick, 2008 ▶); software used to prepare material for publication: *SHELXTL*.

## Supplementary Material

Crystal structure: contains datablocks I, global. DOI: 10.1107/S1600536811003527/vm2076sup1.cif

Structure factors: contains datablocks I. DOI: 10.1107/S1600536811003527/vm2076Isup2.hkl

Additional supplementary materials:  crystallographic information; 3D view; checkCIF report

## Figures and Tables

**Table 1:** Hydrogen-bond geometry (Å, °)

*D*—H⋯*A*	*D*—H	H⋯*A*	*D*⋯*A*	*D*—H⋯*A*
N—H0*A*⋯O^i^	0.86	2.10	2.912 (2)	158

Symmetry code: (i) 
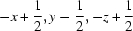
.

**Fig. 1.**
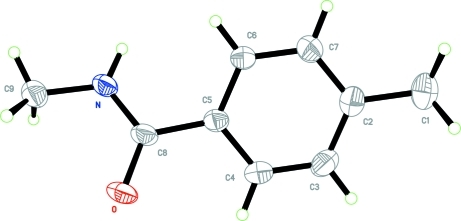
The molecular structure of (I), with the atom-numbering scheme. Displacement ellipsoids are drawn at the 50% probability level.

**Fig. 2.**
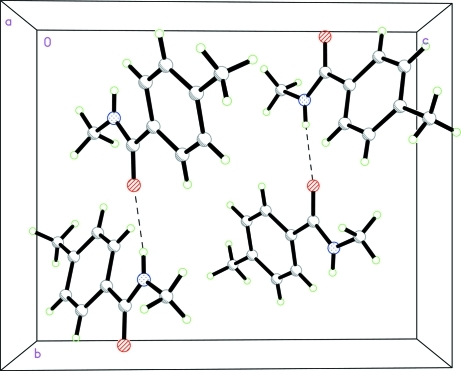
Packing diagram for (I) showing the N—H···O hydrogen bonds as dashed lines.

## supplementary crystallographic information

### Comment

Benzamide derivatives exhibit interesting biological activities such as antibacterial and antifungal effects (Lee *et al.*, 2009) We report here the crystal structure of the title compound *N*,4-dimethylbenzamide (I), an important organic intermediate (Fig. 1). Bond lengths and angles are within normal ranges (Allen *et al.*, 1987).

In the crystal packing of (I) the molecules are connected together *via* N—H···O intermolecular hydrogen bonds to form a one-dimensional network in the *b* direction (Table 1, graph set C1,1(4)), which seems to be very effective in the stabilization of the crystal structure.

### Experimental

The title compound, (I) was prepared by a method reported in literature (Lee *et al.* (2009)). Crystals were obtained by dissolving (I) (0.2 g, 1.34 mmol) in ethanol (25 ml) and evaporating the solvent slowly at room temperature for about 7 d.

### Refinement

All H atoms were positioned geometrically and constrained to ride on their parent atoms, with C—H = 0.93 Å for aromatic H, 0.96 Å for methyl H and 0.86 Å for N—H, respectively. The *U*_iso_(H) = *xU*_eq_(C), where *x* = 1.2 for aromatic H and N—H, and *x* = 1.5 for methyl H.

### Crystal data

**Table:**

C_9_H_11_NO	*F*(000) = 320
*M**_r_* = 149.19	*D*_x_ = 1.205 Mg m^−^^3^
Monoclinic, *P*2_1_/*n*	Mo *K*α radiation, λ = 0.71073 Å
Hall symbol: -P 2yn	Cell parameters from 25 reflections
*a* = 6.7670 (14) Å	θ = 9–13°
*b* = 9.946 (2) Å	µ = 0.08 mm^−^^1^
*c* = 12.229 (2) Å	*T* = 293 K
β = 92.63 (3)°	Block, colourless
*V* = 822.2 (3) Å^3^	0.30 × 0.20 × 0.10 mm
*Z* = 4	

### Data collection

**Table:**

Enraf–Nonius CAD-4 diffractometer	1062 reflections with *I* > 2σ(*I*)
Radiation source: fine-focus sealed tube	*R*_int_ = 0.033
graphite	θ_max_ = 25.4°, θ_min_ = 2.6°
ω/2θ scans	*h* = 0→8
Absorption correction: ψ scan (North *et al.*, 1968)	*k* = −11→11
*T*_min_ = 0.977, *T*_max_ = 0.992	*l* = −14→14
3362 measured reflections	3 standard reflections every 200 reflections
1510 independent reflections	intensity decay: 1%

### Refinement

**Table:**

Refinement on *F*^2^	Secondary atom site location: difference Fourier map
Least-squares matrix: full	Hydrogen site location: inferred from neighbouring sites
*R*[*F*^2^ > 2σ(*F*^2^)] = 0.050	H-atom parameters constrained
*wR*(*F*^2^) = 0.166	*w* = 1/[σ^2^(*F*_o_^2^) + (0.1*P*)^2^ + 0.080*P*] where *P* = (*F*_o_^2^ + 2*F*_c_^2^)/3
*S* = 1.01	(Δ/σ)_max_ < 0.001
1510 reflections	Δρ_max_ = 0.20 e Å^−^^3^
103 parameters	Δρ_min_ = −0.15 e Å^−^^3^
0 restraints	Extinction correction: *SHELXL97* (Sheldrick, 2008), Fc^*^=kFc[1+0.001xFc^2^λ^3^/sin(2θ)]^-1/4^
Primary atom site location: structure-invariant direct methods	Extinction coefficient: 0.028 (8)

### Special details

**Table:**

Geometry. All e.s.d.'s (except the e.s.d. in the dihedral angle between two l.s. planes) are estimated using the full covariance matrix. The cell e.s.d.'s are taken into account individually in the estimation of e.s.d.'s in distances, angles and torsion angles; correlations between e.s.d.'s in cell parameters are only used when they are defined by crystal symmetry. An approximate (isotropic) treatment of cell e.s.d.'s is used for estimating e.s.d.'s involving l.s. planes.
Refinement. Refinement of *F*^2^ against ALL reflections. The weighted *R*-factor *wR* and goodness of fit *S* are based on *F*^2^, conventional *R*-factors *R* are based on *F*, with *F* set to zero for negative *F*^2^. The threshold expression of *F*^2^ > σ(*F*^2^) is used only for calculating *R*-factors(gt) *etc*. and is not relevant to the choice of reflections for refinement. *R*-factors based on *F*^2^ are statistically about twice as large as those based on *F*, and *R*- factors based on ALL data will be even larger.

### Fractional atomic coordinates and isotropic or equivalent isotropic displacement parameters (Å^2^)

**Table:**

	*x*	*y*	*z*	*U*_iso_*/*U*_eq_	
O	0.2041 (3)	0.99892 (14)	0.23558 (16)	0.0773 (6)	
N	0.2849 (2)	0.78998 (15)	0.28900 (14)	0.0514 (5)	
H0A	0.2554	0.7059	0.2868	0.062*	
C1	−0.5644 (4)	0.6995 (3)	0.0146 (2)	0.0766 (8)	
H1A	−0.6295	0.6353	0.0592	0.115*	
H1B	−0.5367	0.6591	−0.0543	0.115*	
H1C	−0.6485	0.7763	0.0023	0.115*	
C2	−0.3742 (3)	0.7431 (2)	0.07191 (17)	0.0550 (6)	
C3	−0.2949 (4)	0.8698 (2)	0.05499 (19)	0.0652 (7)	
H3A	−0.3599	0.9283	0.0062	0.078*	
C4	−0.1226 (3)	0.9106 (2)	0.10867 (18)	0.0591 (6)	
H4A	−0.0750	0.9968	0.0969	0.071*	
C5	−0.0184 (3)	0.82556 (17)	0.18015 (15)	0.0440 (5)	
C6	−0.0961 (3)	0.69834 (18)	0.19627 (17)	0.0530 (6)	
H6A	−0.0291	0.6387	0.2434	0.064*	
C7	−0.2701 (3)	0.6593 (2)	0.14374 (18)	0.0581 (6)	
H7A	−0.3195	0.5738	0.1569	0.070*	
C8	0.1657 (3)	0.87739 (18)	0.23661 (16)	0.0479 (5)	
C9	0.4623 (3)	0.8321 (2)	0.3494 (2)	0.0613 (6)	
H9A	0.5168	0.7575	0.3904	0.092*	
H9B	0.4309	0.9034	0.3987	0.092*	
H9C	0.5571	0.8637	0.2993	0.092*	

### Atomic displacement parameters (Å^2^)

**Table:**

	*U*^11^	*U*^22^	*U*^33^	*U*^12^	*U*^13^	*U*^23^
O	0.0747 (11)	0.0273 (8)	0.1272 (15)	−0.0054 (7)	−0.0245 (10)	0.0023 (8)
N	0.0498 (10)	0.0296 (8)	0.0737 (12)	−0.0025 (7)	−0.0084 (8)	−0.0006 (7)
C1	0.0590 (15)	0.0951 (19)	0.0743 (16)	−0.0004 (13)	−0.0132 (12)	−0.0086 (14)
C2	0.0481 (12)	0.0610 (13)	0.0555 (12)	0.0047 (10)	−0.0017 (10)	−0.0086 (10)
C3	0.0654 (15)	0.0563 (13)	0.0721 (15)	0.0125 (11)	−0.0159 (12)	0.0065 (11)
C4	0.0631 (14)	0.0396 (11)	0.0739 (14)	0.0040 (10)	−0.0038 (12)	0.0083 (10)
C5	0.0446 (11)	0.0321 (9)	0.0552 (11)	0.0050 (8)	0.0007 (9)	−0.0030 (8)
C6	0.0531 (12)	0.0351 (10)	0.0695 (13)	0.0000 (9)	−0.0108 (10)	0.0047 (9)
C7	0.0547 (13)	0.0463 (12)	0.0724 (14)	−0.0055 (9)	−0.0068 (11)	−0.0010 (10)
C8	0.0503 (12)	0.0288 (9)	0.0647 (12)	0.0016 (8)	0.0017 (9)	−0.0024 (8)
C9	0.0537 (13)	0.0506 (12)	0.0783 (15)	−0.0037 (10)	−0.0122 (11)	−0.0019 (11)

### Geometric parameters (Å, °)

**Table:**

O—C8	1.237 (2)	C3—H3A	0.9300
N—C8	1.330 (2)	C4—C5	1.386 (3)
N—C9	1.442 (3)	C4—H4A	0.9300
N—H0A	0.8600	C5—C6	1.388 (3)
C1—C2	1.501 (3)	C5—C8	1.489 (3)
C1—H1A	0.9600	C6—C7	1.372 (3)
C1—H1B	0.9600	C6—H6A	0.9300
C1—H1C	0.9600	C7—H7A	0.9300
C2—C7	1.381 (3)	C9—H9A	0.9600
C2—C3	1.389 (3)	C9—H9B	0.9600
C3—C4	1.373 (3)	C9—H9C	0.9600
			
C8—N—C9	121.90 (17)	C4—C5—C6	117.46 (19)
C8—N—H0A	119.0	C4—C5—C8	118.17 (17)
C9—N—H0A	119.0	C6—C5—C8	124.35 (17)
C2—C1—H1A	109.5	C7—C6—C5	120.96 (19)
C2—C1—H1B	109.5	C7—C6—H6A	119.5
H1A—C1—H1B	109.5	C5—C6—H6A	119.5
C2—C1—H1C	109.5	C6—C7—C2	121.9 (2)
H1A—C1—H1C	109.5	C6—C7—H7A	119.0
H1B—C1—H1C	109.5	C2—C7—H7A	119.0
C7—C2—C3	117.0 (2)	O—C8—N	121.39 (19)
C7—C2—C1	121.6 (2)	O—C8—C5	120.36 (18)
C3—C2—C1	121.4 (2)	N—C8—C5	118.24 (16)
C4—C3—C2	121.5 (2)	N—C9—H9A	109.5
C4—C3—H3A	119.2	N—C9—H9B	109.5
C2—C3—H3A	119.2	H9A—C9—H9B	109.5
C3—C4—C5	121.2 (2)	N—C9—H9C	109.5
C3—C4—H4A	119.4	H9A—C9—H9C	109.5
C5—C4—H4A	119.4	H9B—C9—H9C	109.5
			
C7—C2—C3—C4	−1.1 (3)	C3—C2—C7—C6	−0.1 (3)
C1—C2—C3—C4	178.9 (2)	C1—C2—C7—C6	179.9 (2)
C2—C3—C4—C5	1.5 (4)	C9—N—C8—O	1.5 (3)
C3—C4—C5—C6	−0.7 (3)	C9—N—C8—C5	−177.53 (18)
C3—C4—C5—C8	−179.17 (19)	C4—C5—C8—O	13.1 (3)
C4—C5—C6—C7	−0.4 (3)	C6—C5—C8—O	−165.2 (2)
C8—C5—C6—C7	177.91 (18)	C4—C5—C8—N	−167.81 (18)
C5—C6—C7—C2	0.8 (3)	C6—C5—C8—N	13.8 (3)

### Hydrogen-bond geometry (Å, °)

**Table:**

*D*—H···*A*	*D*—H	H···*A*	*D*···*A*	*D*—H···*A*
N—H0A···O^i^	0.86	2.10	2.912 (2)	158

Symmetry codes: (i) −*x*+1/2, *y*−1/2, −*z*+1/2.

## References

[bb1] Allen, F. H., Kennard, O., Watson, D. G., Brammer, L., Orpen, A. G. & Taylor, R. (1987). *J. Chem. Soc. Perkin Trans. 2*, pp. S1–19.

[bb2] Enraf–Nonius (1985). *CAD-4 Software* Enraf–Nonius, Delft, The Netherlands.

[bb3] Harms, K. & Wocadlo, S. (1995). *XCAD4* University of Marburg, Germany.

[bb4] Lee, S., Song, K. H., Choe, J., Ju, J. & Jo, Y. (2009). *J. Org. Chem.* **74**, 6358–6361. 10.1021/jo901065y 19572571

[bb5] North, A. C. T., Phillips, D. C. & Mathews, F. S. (1968). *Acta Cryst.* A**24**, 351–359.

[bb6] Sheldrick, G. M. (2008). *Acta Cryst.* A**64**, 112–122. 10.1107/S0108767307043930 18156677

